# Transferable spectral models for high-throughput estimation of winter wheat net photosynthetic rate: a cross-scale approach using leaf and UAV hyperspectral data

**DOI:** 10.3389/fpls.2026.1948151

**Published:** 2026-09-03

**Authors:** Menglei Dai, Limin Gu, Baoyuan Zhang, Xiaoyuan Bao, Ying Ren, Shuping Xiong, Wenchao Zhen, Xiaohe Gu

**Affiliations:** 1 State Key Laboratory of North China Crop Improvement and Regulation/College of Agronomy, Hebei Agricultural University/Key Laboratory of North China Water saving Agriculture, Ministry of Agriculture and Rural Affairs, Baoding, Hebei, China; 2 Research Center of Information Technology, Beijing Academy of Agriculture and Forestry Sciences, Beijing, China; 3 College of Agriculture, Nanjing Agricultural University, Nanjing, China; 4 State Key Laboratory of Aridland Crop Science, Gansu Agriculture University, Lanzhou, Gansu, China; 5 College of Plant Protection, Hebei Agricultural University, Baoding, Hebei, China; 6 College of Agronomy, Henan Agricultural University, Zhengzhou, Henan, China

**Keywords:** hyperspectral, machine learning, net photosynthetic rate, sensitive feature, UAV, winter wheat

## Abstract

**Introduce:**

Net photosynthetic rate (NPR) is a key indicator of photosynthetic capacity that directly governs the production and accumulation of biomass. Non-destructive sensing techniques based on multi-scale sensors (e.g. field spectroradiometers and UAV platforms) face challenges in simultaneously achieving high accuracy, strong transferability, and high-throughput monitoring.

**Methods:**

This study addresses the urgent need for cross-scale extension of spectral models to bridge this methodological gap. We focused on estimating the NPR of winter wheat using leaf hyperspectral reflectance and extending the estimation model to a UAV platform by statistically characterizing and correcting spectral discrepancies between field spectroradiometer and UAV hyperspectral measurements. At first, the raw spectral reflectance was transformed into relevant vegetation indices and extracted sensitive features using Competitive Adaptive Reweighted Sampling (CARS) and Successive Projections Algorithm (SPA). Then, estimation models for the NPR were constructed using Random Forest (RF) and Partial Least Squares Regression (PLSR) methods. Finally, the performance of the eight estimation models was compared using coefficient of determination (R^2^) and Root Mean Square Error (RMSE). The UAV images from different growing seasons were input into the optimal model to generate spatial monitoring maps.

**Results:**

The results showed that transforming raw spectral reflectance into vegetation indices significantly improved model performance. RF exhibited notably higher accuracy than PLSR, with the VI-SPARF model achieving the best predictive performance. Through effective calibration, the system achieved cross-scale application from ground-based spectrometers to UAV hyperspectral imaging.

**Conclusion:**

Overall, this study confirms the robustness and transferability of spectral-physiological relationships across temporal scales and sensing platforms, providing methodological support for operational, high throughput monitoring of crop photosynthesis.

## 1 Introduction

The Wheat is one of the three staple food crops in the world, covering an estimated 20 billion hectares of land, and its production has a significant impact on global food security concerns. Photosynthesis plays a crucial role in vegetative and reproductive growth of winter wheat as derived more than 95% of dry matter, and highly related with grain yield (Gaju et al., 2016). The net photosynthetic rate (NPR) is a crucial indicator for measuring the photosynthetic capacity of winter wheat (Li et al., 2023). Estimating the NPR of winter wheat is of great significance for mastering the dynamics of growth, optimizing varieties, and adjusting water and fertilizer supplies.

Traditional methods for measuring the NPR rely on gas exchange measurements in the field. This method is accurate but time and labor consuming, and unsuitable for high-throughput measurements in the field (Meacham-Hensold et al., 2020; Yuan et al., 2023). Additionally, the results cannot provide other phenotypic characteristics information (Heckmann et al., 2017). The hyperspectral technology provide a nondestructive, rapid, and cost-effective method of identifying the phenotypic traits of individual plants (Finkel, 2009; Schäfer et al., 2015). This method enables rapid data acquisition over short time intervals and supports long-term monitoring of dynamic physiological traits in crops. With the advancement of remote sensing technologies in agriculture, reflectance spectroscopy has emerged as a valuable tool for predicting crop physiological activity (Serbin et al., 2012). Specifically, leaf spectral reflectance represents a promising high-throughput approach for estimating the photosynthetic capacity in crops.

Many approaches have been developed for rapidly and non-destructively assessing this capacity using leaf spectral reflectance (Serbin et al., 2012; Ainsworth et al., 2014). Partial least squares regression (PLSR) is a commonly used linear model to estimate photosynthetic capacity. PLSR models for kale and maize have already been developed and have demonstrated high accuracy in estimating photosynthetic capacity (Heckmann et al., 2017). However, due to the heterogeneity of PLSR models for assessing physiological activity in crops (Fu et al., 2019), it remains to be investigated whether this model is applicable to other crops. Mathematical modifications and vegetation index transformations are mostly utilized for raw spectral reflectance to estimate the NPR of winter wheat. Mathematical transformations include inverse, logarithmic, and first-order derivative transformations. However, there is a phenomenon in which models constructed using mathematical transformations perform worse than models constructed using raw reflectance data (Endo et al., 2001). The processing of raw reflectance data has a direct impact on the final estimation of the model, and vegetation indices can be applied to the raw data, in addition to mathematical transformations. Many hyperspectral vegetation indices are currently available for estimating winter wheat net photosynthesis rate (Tan et al., 2018; Zhou et al., 2021). When selecting a vegetation index, it is important to consider the relationship between the selected index’s spectral bands and the net photosynthesis rate from a crop science perspective (focusing on indices based on visible bands). For instance, the MCARI is a widely-used vegetation index with a precision of 0.90 on estimating net photosynthesis rate (El-Hendawy et al., 2017). Compared to evaluating the predictive ability of individual vegetation indices, constructing models by combining these indices is also a reliable method. It has been shown that models constructed by combining relevant hyperspectral vegetation indices outperform those constructed with a single vegetation index (Zhou et al., 2021). This approach provides valuable insights for our subsequent research on estimating NPR of winter wheat.

With continued advances in sensing technology, a wide range of spectral monitoring sensors has been developed to meet diverse application requirements. Field spectroradiometers provide accurate and non-destructive measurements of crop physiological traits. They are closely related to crop physiological activity and nutritional status and facilitate the interpretation of spectral response mechanisms. However, ground-based measurements cannot meet the high throughput monitoring demands of modern precision agriculture. UAV-based retrieval of crop phenotypic traits has become increasingly mature and enables flexible and high-throughput monitoring. However, winter wheat has dense canopies and strong spatial heterogeneity. Spectral signals are also affected by cultivar characteristics and soil-related environmental factors. As a result, models developed directly from UAV data often show limited stability, which restricts their transferability. Previous studies have attempted to integrate information from ground-based and UAV hyperspectral data to improve winter wheat yield estimation (Feng et al., 2022). Although encouraging improvements were reported, true cross-scale extension was not fully achieved. To address these limitations, this study develops a spectral estimation model for NPR using field spectroradiometer data. The model is then extended to the UAV platform by statistically characterizing and correcting spectral discrepancies between ground-based and UAV hyperspectral measurements. Due to the heterogeneity of PLSR models in estimating crop physiological traits (Fu et al., 2019), this study incorporated Random Forest (RF) as a non-linear alternative and comparatively evaluated their respective performances for NPR estimation. The specific objectives of this study were as follows: (1) to compare different feature extraction methods and optimize hyperspectral dimensionality reduction; (2) to construct an estimation model for winter NPR based on the selected sensitive feature combinations; (3) to assess the accuracy and robustness of the constructed model using independent testing datasets.

## 2 Materials and methods

### 2.1 Experimental design

**Figure 1:**
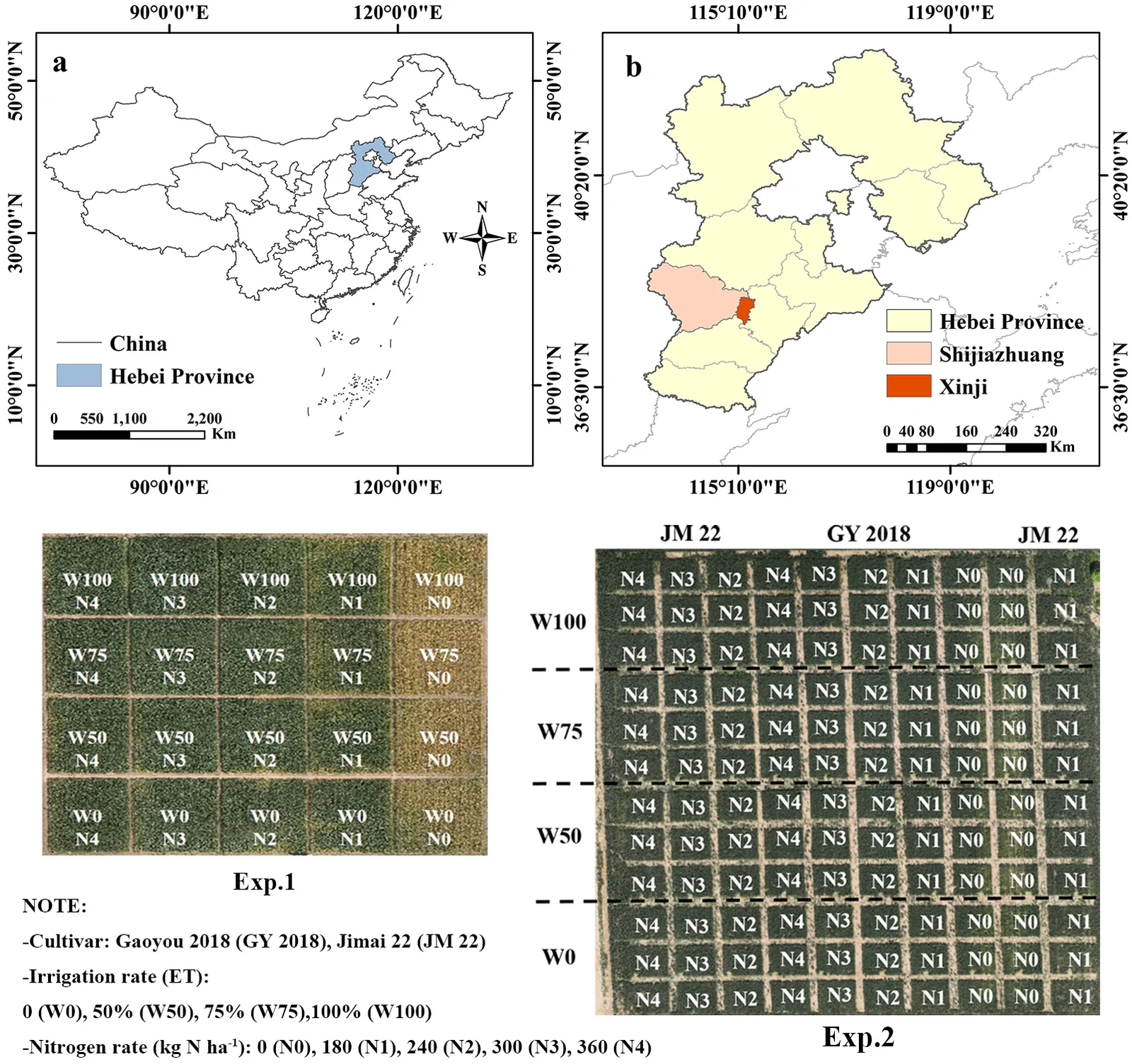
Geographic location of the regional experimental sites.

The experiment was conducted in Xinji City (33°24’N, 114°44’E), Hebei Province, China, which falls within the warm temperate semi-humid monsoon continental climate zone and experiences distinct seasons. The cropping system follows a biannual cropping regime, typical of the wheat-maize rotation areas in the central plains of Hebei. The experiments covered the two growing seasons of winter wheat: Exp.1 from October 2021 to June 2022 and Exp.2 from October 2023 to June 2024 (**Figure 1**). Two-factor orthogonal treatments was set, with four levels of irrigation amount (W) and five levels of nitrogen application rate (N). The irrigation treatments were based on evapotranspiration (ET) and included: fully irrigation (100% ET), slight drought (75% ET), moderate drought (50% ET), and severe drought (0% ET), labeled as W100, W75, W50, and W0, respectively. The nitrogen application levels were 0, 180, 240, 300, 360 kg N hm-2, labeled as N0, N1, N2, N3 and N4, respectively. Other field management were the same as those used in conventional high-yielding fields.

### 2.2 Data collection

#### 2.2.1 NPR measurement

The LI-6800 photosynthesis system manufactured by LI-COR was utilized to measure the NPR of top leaves of each plant. During the measurement process, a circular fluorescence chamber (6800-01F) with an area of 6 cm^2^ was selected, and a CO^2^ cylinder was used to ensure a consistent and stable CO^2^ concentration within the chamber. Additionally, stable factors such as light intensity, humidity, and temperature were set within the chamber. After the plants had adapted to the chamber environment, a stable NPR was obtained. Each leaf was measured three times, and the average value was taken as the NPR of each leaf. Measurements were conducted on clear, cloudless days from 10 a.m. to 12 p.m. and avoid the midday period, which could reduce the physiological activity of winter wheat due to high air temperatures and low relative humidity, potentially affecting the measurements.

#### 2.2.2 Hyperspectral reflectance collection

The collection of hyperspectral data was undertaken on the same day as the acquisition of photosynthesis data. Exp.1 collected leaf hyperspectral reflectance using HR-1024i (SVC, the United States), while Exp.2 collected canopy reflectance using a DJI M300 UAV equipped with X20 Pro hyperspectral camera (Cubert GmbH, Germany) (**Table 1**).

**Table 1:** Parameters of hyperspectral cameras.

Exp.1				
wavelength range	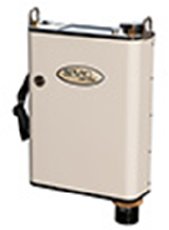	350–1000 nm	1000–1900 nm	1900–2500 nm
bandwidth		1.5 nm	3.6 nm	2.5 nm
spectral resolution		3.0 nm	9.5 nm	6.5 nm
Noise Equivalent Radiance		0.8 x 10–^9^ W	1.2 x 10–^9^ W	1.2 x 10–^9^ W

Exp.2				
model	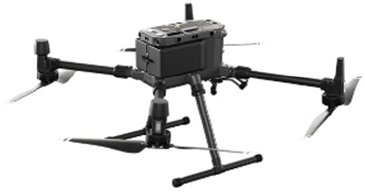 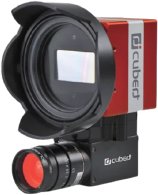	M300 RTK	model	X20 pro
flight time		10:00-14:00	wavelength range	350–1002 nm
flight altitude		50 m	bandwidth	4 nm
flight speed		6.7 m/s	spectral resolution	10 nm
flight heading		north-south	overlap ratio	75%
PTZ angle		-90°	image resolution	1886*1886

During Exp.1, spectral calibration was performed using a standard white reference panel before measurements. Individual leaves were clamped at the middle position, with five replicate measurements acquired per leaf. For Exp.2, under stable sunlight conditions, the lens was adjusted to ensure that the calibration panel fully occupied the field of view without shadow interference, followed by white reference calibration. The lens was then covered to block all light for dark current calibration. The collected images underwent radiometric calibration for white balance and dark current using Cubert Utils Touch software. Subsequently, the images were stitched and converted to BIL format in ENVI 5.3 to obtain full-band reflectance data.

### 2.3 Modeling methods for NPR

This study investigated the flag leaves of winter wheat plants under varying water and fertilizer supply conditions. Sensitive feature variables were extracted using Competitive Adaptive Reweighted Sampling and Successive Projections Algorithm based on the raw hyperspectral reflectance of the leaves and corresponding combinations of vegetation indices. Estimation models were constructed using both the Random Forest and Partial Least Squares Regression methods. The applicability and feasibility of each model were analyzed to determine the best estimation model.

#### 2.3.1 Processing of raw spectral reflectance

For the raw spectral reflectance obtained directly by the ground object spectrometer, after removing outliers from five repeated measurements of each leaf, the average value is calculated as the spectral reflectance data, and draw the spectral reflectivity curve. The correlation between reflectivity and NPR in each band was analyzed to verify the correctness of data acquisition. Based on previous research on vegetation indices associated to winter wheat NPR, the processed raw spectral reflectance is transformed using the vegetation index.

A total of 33 vegetation indices were chosen (**Table 2**), which have the potential to evaluate vegetation parameters related to physiology, morphology, and biochemistry.

**Table 2:** Vegetation indices used in this study.

Full name & Abbr	Formula	Source
Difference index (DI)	R_800_ -R_500_	(Gobron et al., 2000)
Difference vegetation index (DVI)	R_800_ -R_680_	(Gobron et al., 2000)
Normalized difference index (NDI)	(R_800_ -R_680_)/(R_800_ +R_680_)	(Gonzalez-Dugo et al., 2015)
Normalized difference vegetation index (NDVI)	(R_800_ -R_670_)/(R_800_ +R_670_)	(Rouse et al., 1974)
Normalized pigments chlorophyll ratio index (NPCI)	(R_680_ -R_430_)/(R680+R_430_)	(Ridao et al., 1998)
Transformed chlorophyll absorption in reflectance index (TCARI)	3 *[(R_700_ -R_670_)-0.2*(R_700_ -R_550_)*(R_700_/R_670_)]	(Guo et al., 2015)
Red green ratio (RGR)	(R_612_ +R_660_)/(R_510_ +R_560_)	(Steven, 1998)
Visible atmospherically resistant index (VARI)	(R_555_ -R_680_)/(R_555_ +R_680_ -R_480_)	(Qi et al., 1994)
Green normalized difference vegetation index (GNDVI)	(R_800_ -R_550_)/(R_800_ +R_550_)	(Wang et al., 2009)
Pigment specific normalized difference 1 (PSND[800,635])	(R_800_ -R_635_)/(R_800_ +R_635_)	(Bargain et al., 2013)
Pigment specific normalized difference 2 (PSND[800,470])	(R_800_ -R_470_)/(R_800_ +R_470_)	(Bargain et al., 2013)
Ratio vegetation index (RVI)	R_800_/R_670_	(Jordan, 1969)
Enhanced vegetation index (EVI)	2.5*(R_860_ -R_680_)/(R_800_ + 6*R_680_ -7.5*R_450_ +1.0)	(Huete et al., 1994)
Greenness index (GI)	R_554_/R_667_	(Zarco-Tejada et al., 2005)
Red edge model (CI_730)	R_800_/R_730_ -1.0	(Mehdaoui and Anane, 2020)
Red edge model (CI_709)	R_800_/R_709_ -1.0	(Mehdaoui and Anane, 2020)
Chrollophy index at green band (ch1green)	R_800_/R_500_ -1.0	(Gitelson et al., 2006)
Normalized difference red edge (NDRE)	(R_790_ - R_720_)/(R_790_ +R_720_)	(Fitzgerald et al., 2010)
Chlorophyll_a absorption ratio index (CARI_a)	(R_700_ -R_550_)/150	(Kim et al., 1994)
Chlorophyll_b absorption ratio index (CARI_b)	R_550_ -CARI_a*500	(Kim et al., 1994)
MERIS terrestrial chlorophyll index (MTCI)	(R_750_ -R_710_)(R_710_ -R_680_)	(Chen, 1996)
Photochemical reflectance index improved (PRI_2)	(R_528_ -R_567_)/(R_528_ +R_567_)	(Gamon et al., 1992)
Moisture stress index (MSI)	R_1600_/R_820_	(Hunt and Rock, 1989)
Water index (WI)	R_900_/R_970_	(Penuelas et al., 1997)
Normalized multi-band drought index (NMDI)	((R_860_ +R_1640_ +R_2130_) /(R_860_ +R_1640_ -R_2130_)	(Fitzgerald et al., 2010)
Global vegetation moisture index (GVMI)	(R_820_ +0.1 - R_1640_ -0.02)/(R_820_ +0.1+R_1640_ +0.02)	(Ceccato et al., 2002)
Normalized difference water index (NDWI_1200)	(R_886_ -R_1200_)/(R_886_ +R_1200_)	(Gao, 1996)
Normalized difference water index (NDWI_1240)	(R_886_ -R_1240_)/(R_886_ +R_1240_)	(Gao, 1996)
Normalized difference water index (NDWI_1640)	(R_886_ -R_1640_)/(R_886_ +R_1640_)	(Gao, 1996)
Modified chlorophyll absorption in reflectance index (MCARI)	[(R_700_ -R_670_)-0.2*(R_700_ -R_550_)] *(R_700_/R_670_)	(Major et al., 1990)
Photochemical reflectance index (PRI)	(R_531_ -R_570_)/(R_530_ +R_570_)	(Dash and Curran, 2004)
Structure intensive pigment index (SIPI)	(R_800_ -R_445_)(R_800_ -R_680_)	(Chen, 1996)
Red and green vegetation index (RGVI)	R_550_/R_670_	(Jordan, 1969)

#### 2.3.2 Methods of extracting feature variables

Competitive Adaptive Reweighted Sampling (CARS) selects sensitive features based on the principle of optimal model accuracy. In this study, a subset of wavelengths was selected in order according to the importance of each wavelength in an iterative competition, and the percentage of the absolute value of the regression coefficient on the interpretation of the target variable was calculated in several iterations, and the wavelength in the subset with the smallest root mean square error of cross validation (RMSECV) of the PLS model was selected as the sensitive wavelength. The theoretical basis of this approach is linear regression analysis, and is one of the main approaches to model interpretation. In this study, the CARS algorithm was selected for feature preference of raw reflectance and vegetation indices, and the raw variables were recombined to facilitate the creation of an estimation model.

Successive Projections Algorithm (SPA) is a forward variable selection method that minimizes vector space collinearity. It starts by selecting a wavelength from the raw spectrum and adding a new variable in each iteration until the number of selected variables reaches a set value and the root mean square error (RMSE) is minimized. In this study, the feature variables extracted by the SPA algorithm are used as input variables when building the model. This algorithm can filter the raw spectral data to identify the sensitive wavelengths, which reduces computational complexity and may improve accuracy.

#### 2.3.3 Modelling methods

Random Forest (RF) is a form of integrated learning in which multiple learners are blended and fused to produce a more precise and stable model. Several subsets are extracted from 100 samples, each of which is used to train a corresponding sub-learner. The regression results obtained from these sub learners are then averaged using a simple arithmetic mean to produce the final output. The estimation results from each learner in the forest are tallied, which are used to determine the estimated NPR through a voting method.

Partial Least Squares Regression (PLSR) is a multivariate statistical data analysis method. In this study, we analyzed the principal components of all-band reflectance, then established the regression relationship between these principal components and the NPR. The process is repeated several times to derive additional components until the requirements are met. This ultimately leads to the derivation of a regression expression, which is then subjected to cross-validation checks. A linear model is constructed using PLSR to estimate the NPR. The model takes into account the extraction of principal components from the all band reflectance and the NPR, while maximizing the correlation between the extracted principal components.

##### 2.3.3.1 Partitioning of the sample set

The study employed a grid search algorithm to select the optimal hyperparameters of the model. The data sets, including the training set and the validation set with the 4:1 ratio, were used for five-fold cross-validation. The dataset was randomly divided into a training set and a validation set using the “train_test_split” function in scikit-learn with a fixed random seed (“random_state” function) to avoid subjective selection bias and ensure reproducibility. The model was trained with different combinations of parameters within a specified range, adjusting them in steps to identify the parameters that yielded the highest accuracy on the validation set. It’s a process of training and comparison. Cross-validation is used to evaluate and select models, enabling the selection of the model with the best generalization ability from multiple models. The use of grid search and cross-validation helps to prevent overfitting during the training process and select the most optimal model.

#### 2.3.4 Model accuracy evaluation methods

In this study, the predictive accuracy and stability of all models are evaluated using two metrics: the coefficient of determination (R^2^) and the total root mean square error (RMSE), shown as the following equations.


$$R^{2}=1-\frac{\sum_{i=1}^{n}{\left(y_{i}-{\hat{y}}_{i}\right)}^{2}}{\sum_{i=1}^{n}{\left(y_{i}-{\bar{y}}_{i}\right)}^{2}}\;$$

$$RMSE=\sqrt{\frac{1}{n}\overset{n}{\underset{i=0}{\sum }}{\left(y_{i}-{\bar{y}}_{i}\right)}^{2}}$$

*y_i_* represents the measured value, ${\hat{y}}_{i}$ represents the predicted value, ${\bar{y}}_{i}$ represents the mean of the measured value.

The coefficient of determination (R^2^) is calculated as the square of the correlation coefficient between the predicted and measured values of the model. The higher the R^2^ value, the stronger the performance of the model. On the other hand, the root mean square error (RMSE) is used to quantify the deviation between the estimated and measured values. The smaller the RMSE value, the smaller the deviation between the estimated and measured value.

#### 2.3.5 Expansion and application based on UAV hyperspectral

I. To ensure the reproducibility of research findings and facilitate subsequent predictive applications, this study employed Python’s pickle serialization module for persistent storage of the optimized trained model:

a) File Opening Mode (‘wb’)

The ‘wb’ mode writes data as a binary byte stream, preventing character encoding issues and ensuring the integrity of complex machine learning objects during cross-platform transmission.

b) Object Serialization via pickle.dump()

‘feature_names’: x.columns.tolist(): Simultaneously preserves the list of feature variable names. This ensures that the feature order of input data remains strictly consistent with the training phase, preventing prediction deviations caused by variable misalignment.

c) File Format (.pkl)

The Pickle-specific binary file extension preserves the complete state of class instances, including hyperparameters, fitted estimators (decision tree ensembles), and random states, thereby supporting subsequent model invocation via pickle.load().

II.To mitigate domain shift induced by cross-seasonal phenological variations and cross-platform spectral heterogeneity, a statistical distribution alignment approach was implemented to standardize and rescale UAV-derived vegetation indices to the training domain statistics.

a) Vegetation Index (VI) computation and spectral band matching

The nearest neighbor channel for theoretical wavelengths was located within the UAV hyperspectral data cube (350–1002 nm, 4 nm interval) using arcmin (abs(wavelengths - target)). A band correspondence mapping table (used_bands) was generated to ensure spectral consistency in VI calculation.

b) Feature order consistency assurance

The input VI sequence was strictly enforced through MODEL_FEATURE_ORDER to maintain identical ordering with the x.columns from the training phase, thereby preventing prediction biases arising from feature misalignment.

c) Distribution alignment for cross-domain discrepancy elimination

Statistical Standardization: The VI distribution of UAV imagery (μ_uav,δ_uav)was standardized to a standard normal distribution via z-score normalization:


$$VI_{norm}=\;\frac{VI_{uav}-\;\mu_{uav}}{\delta_{uav}}$$

Distribution Mapping: The standardized data were subsequently rescaled to the statistical distribution of the source domain(μ_train,δ_train):


$$VI_{aligned}=VI_{norm}\times \delta_{train}+\mu_{train}$$

d) Geospatial data processing and map generation

The geospatial projection metadata (profile) of the original UAV imagery was retained using rasterio, ensuring that the output SPAD prediction maps (GeoTIFF format) maintain strict pixel-to-geographic coordinate correspondence with the input images, thereby supporting subsequent GIS-based spatial analysis.

## 3 Results

### 3.1 NPR response to different water and nitrogen supplies

**Figure 2:**
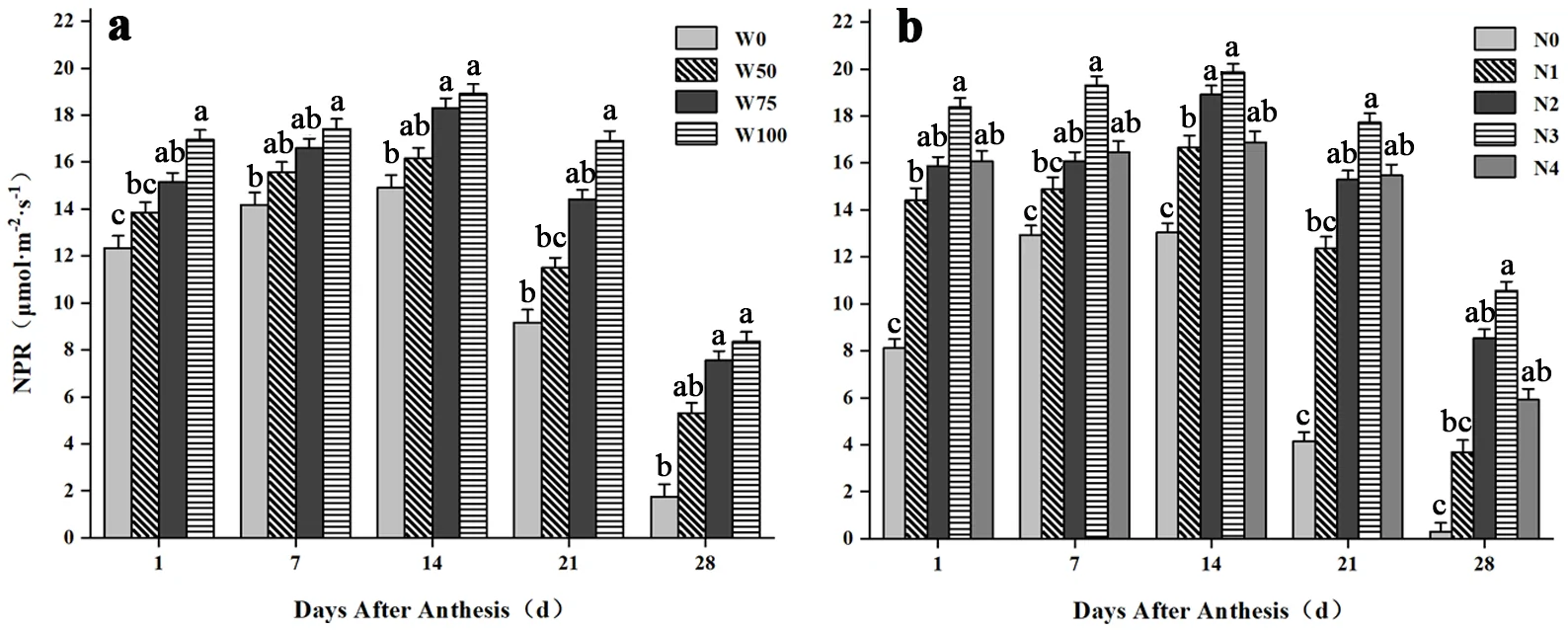
Effect of different irrigation **(a)** and nitrogen application rates **(b)** on NPR. Different lowercase letters indicate significant differences among treatments within the same sampling date (P< 0.05, Tukey’s HSD test).

**Figure 2a** illustrates that irrigation significantly affects the NPR of winter wheat flag leaves, with NPR increasing progressively with the amount of irrigation. It indicates that water deficit significantly inhibits the NPR. **Figure 2b** shows that from N0 to N4, the NPR increases at first and then decreases with the increase of nitrogen ap-plication, and reaches the maximum in the N3 treatment. It indicates that nitrogen supply also has a significant effect on NPR, and excessive nitrogen may cause stress to winter wheat plants. Overall, considering the changes of NPR under different water and nitrogen supplies, from the beginning of the anthesis period to the end of the grain filling period at 28 days after anthesis (DAA), net photosynthesis in-creases at first and then decreases, reached at the peak at 14 DAA, and decreases rapidly after 21 days.

### 3.2 Spectral characteristics of winter wheat leaves under different water and nitrogen supplies

**Figure 3:**
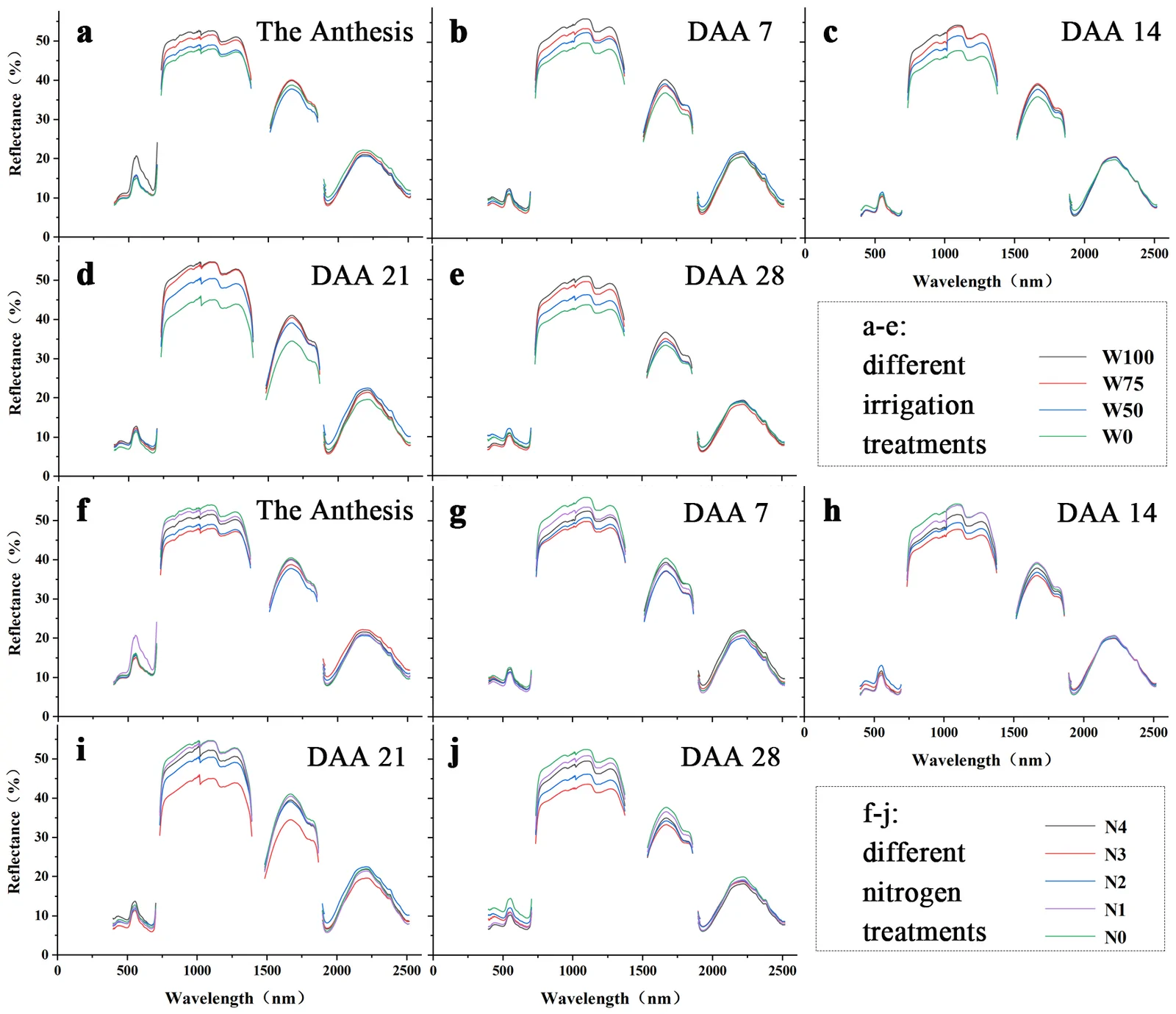
Spectral reflection curve for each water or nitrogen supply condition. **(a-e)** are the spectral characteristics of different irrigation treatments at each growth period, while **(f-j)** are the spectral characteristics of different nitrogen application treatments at each growth period. DAA is the number of days after anthesis and is used to indicate the growth period

The spectral reflectance of winter wheat leaves shows regular changes with different water and nitrogen application rates (**Figure 3**). Within the same growth period, under N3, in the visible band (350–760 nm) and the shortwave infrared band (1420–2500 nm), reflectance de-creases with increasing irrigation, showing a pattern of W0 > W50 > W75 > W100. In contrast, in the near infrared (760–1300 nm), reflectance behaves in the opposite way. For nor-mal irrigation levels, leaf reflectance of the visible band decreases at first and then in-creases with the amount of nitrogen applied, following the pattern N0 > N1 > N4 > N2 > N3, while, the pattern of the near infrared and shortwave near infrared bands is reversed.

The formation of spectral characteristics is closely related to the major components and optical properties of the leaves. For example, the visible band contains weak absorption bands, strong absorption bands and strong reflection peaks of chlorophyll. Variations in chlorophyll content due to water and nitrogen application rates are reflected in this band. In addition, the shortwave infrared band is sensitive to water content, particularly the 1830–2080 nm band, which is a strong absorption band for water. The higher the water content in the leaves, the lower the reflectance in this band.

### 3.3 Sensitive feature selection

Numerous studies have employer vegetation indices (VIs) to assess the NPR of winter wheat [17]. Because different components have different light absorption characteristics, resulting in different reflectance characteristics (such as the strong absorption bands of chlorophyll at 400–450 nm, and those of water vapor and carbon dioxide at 1360–1470 nm), this study selected 33 VIs encompassing three spectral bands: visible (VIS), near infrared (NIR) and shortwave infrared (SWIR). These indices show potential for assessing aspects of plant physiology, morphology and biochemistry.

**Figure 4:**
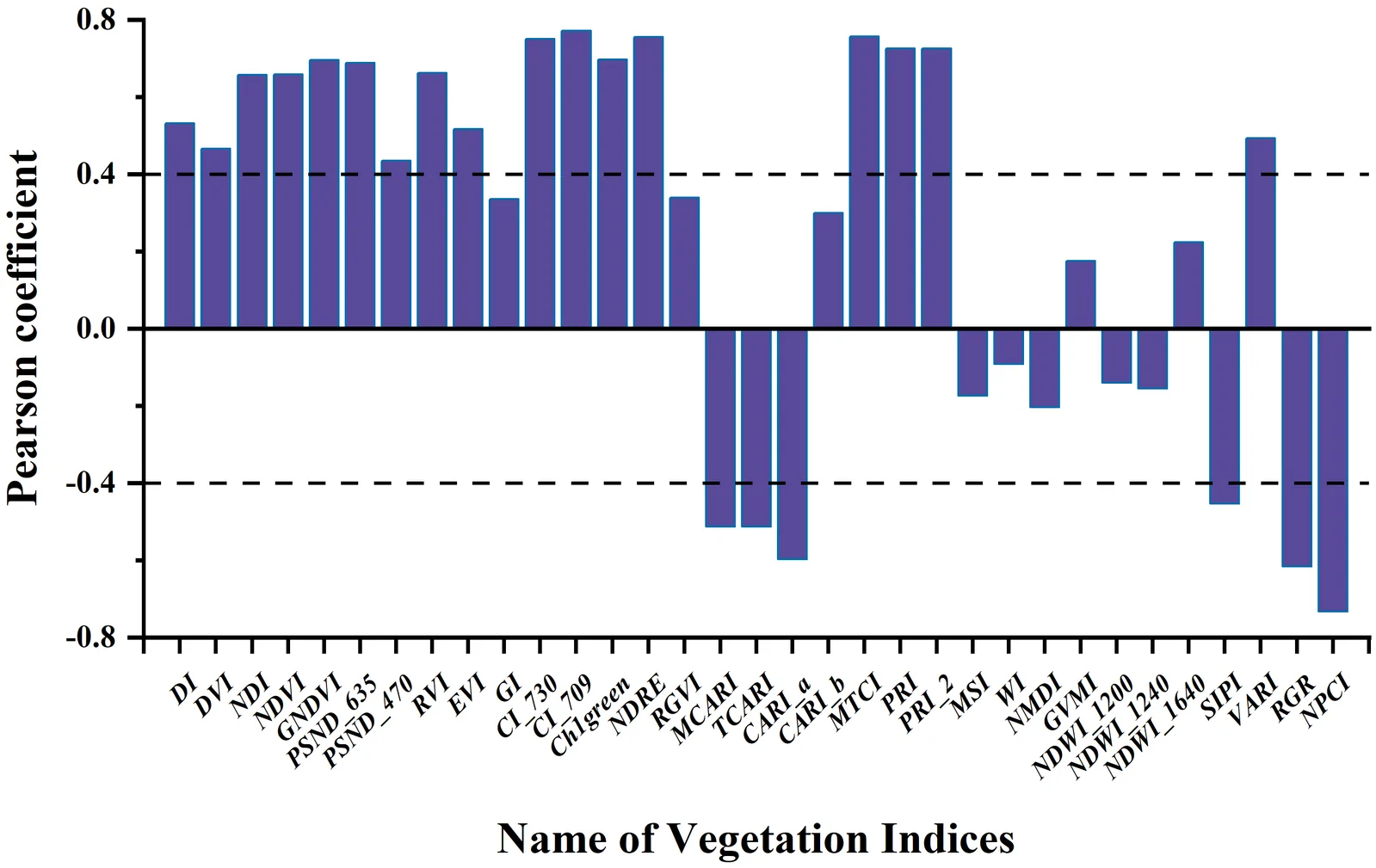
Correlation between vegetation indices and NPR.

Following VI calculation, Pearson correlation analysis was performed against NPR (**Figure 4**). Indices with absolute coefficients below the critical threshold were deemed un-suitable for this study and excluded from mechanistic interpretation. Consequently, 10 VIs were eliminated. The remaining 23 indices were then employed as composite input variables for feature extraction to identify the optimal VI combination.

**Figure 5:**
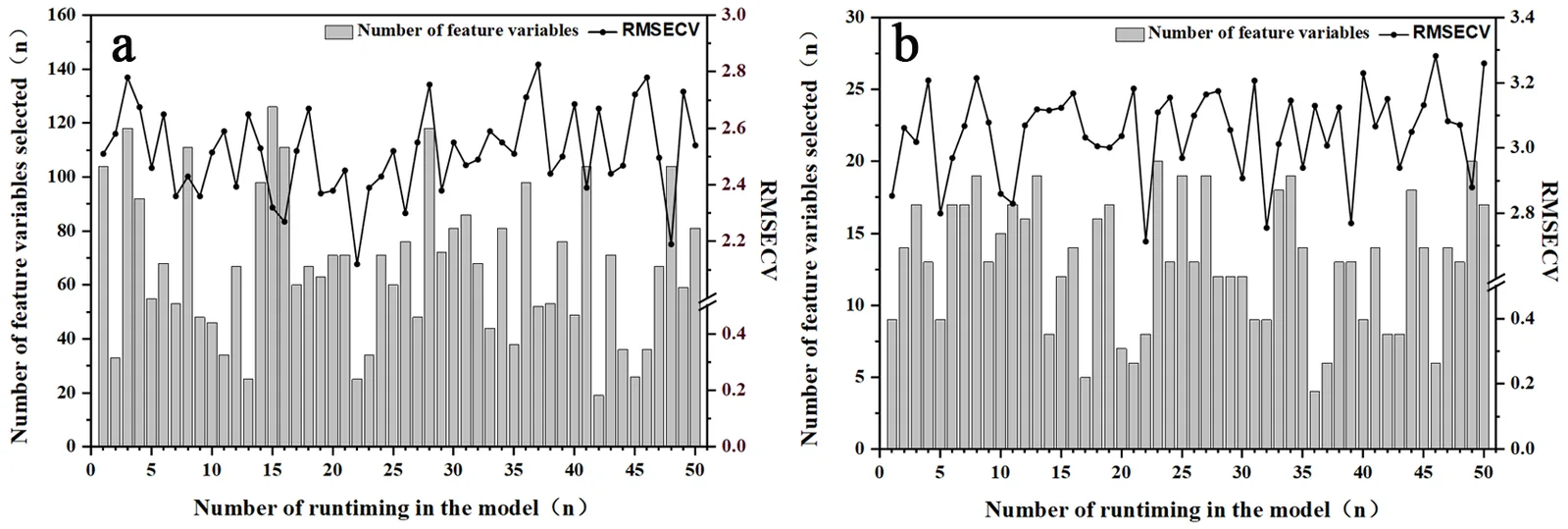
Results of using the CARS algorithm for the raw reflectance **(a)** and VI-combination **(b)**.

As a feature selection algorithm, Competitive Adaptive Reweighted Sampling (CARS) directly selects informative bands from raw spectral data. **Figure 5** shows the evolution of the optimal RMSECV in each iteration over 50 independent runs of the CARS algorithm. The results indicate that despite the CARS algorithm incorporates randomness, the range of variation in the RMSECV values is not significant. Consequently, the variable set de-rived from the 22nd run was selected for subsequent analysis for both full-band reflectance and the vegetation index combination, yielding optimal RMSECV values of 2.120 and 2.714, respectively. The CARS algorithm achieved an effective compression of 2.4% for the full-band reflectance and 34.8% for the vegetation index combination, significantly reducing the number of variables.

**Figure 6:**
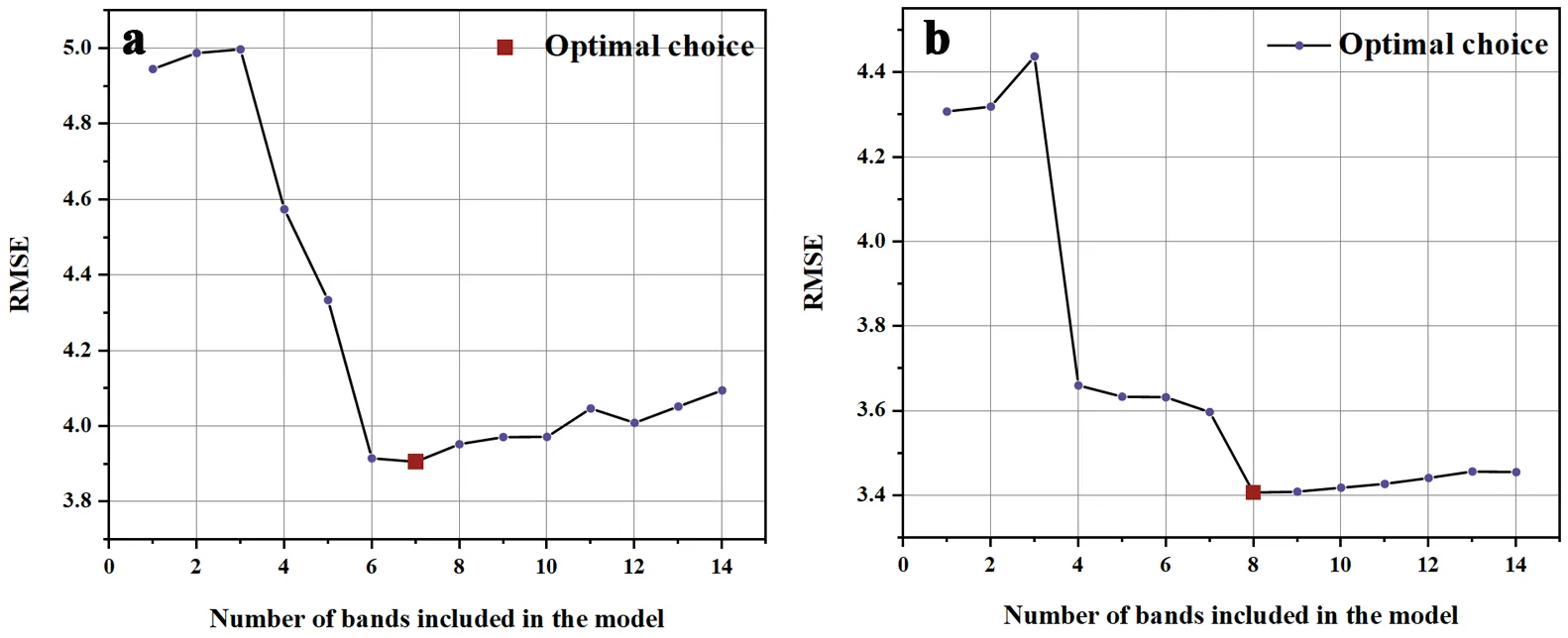
Trend in RMSE during feature extraction by the SPA algorithm for the raw reflectance **(a)** and vegetation index combination **(b)**.

The results of the SPA for band selection on the all-band raw reflectance are shown in **Figure 6a**. The lowest RMSE was obtained when selecting 7 bands with a value of 3.9056. Understanding the principles of the SPA algorithm and its iterative process of band selection, it is clear that this RMSE value represents a local optimum rather than a global optimum. These 7 bands were ultimately selected as the result of the SPA algorithm’s selection on the raw spectral reflectance. Similarly, the results of the SPA algorithm for feature selection on the vegetation index combination are shown in **Figure 6b**, where the RMSE reaches 3.4066 with the selection of 8 bands. The SPA algorithm achieved a dimensionality reduction of 0.7% for the full-band raw reflectance and 34.8% for the 23 vegetation indices.

**Figure 7:**
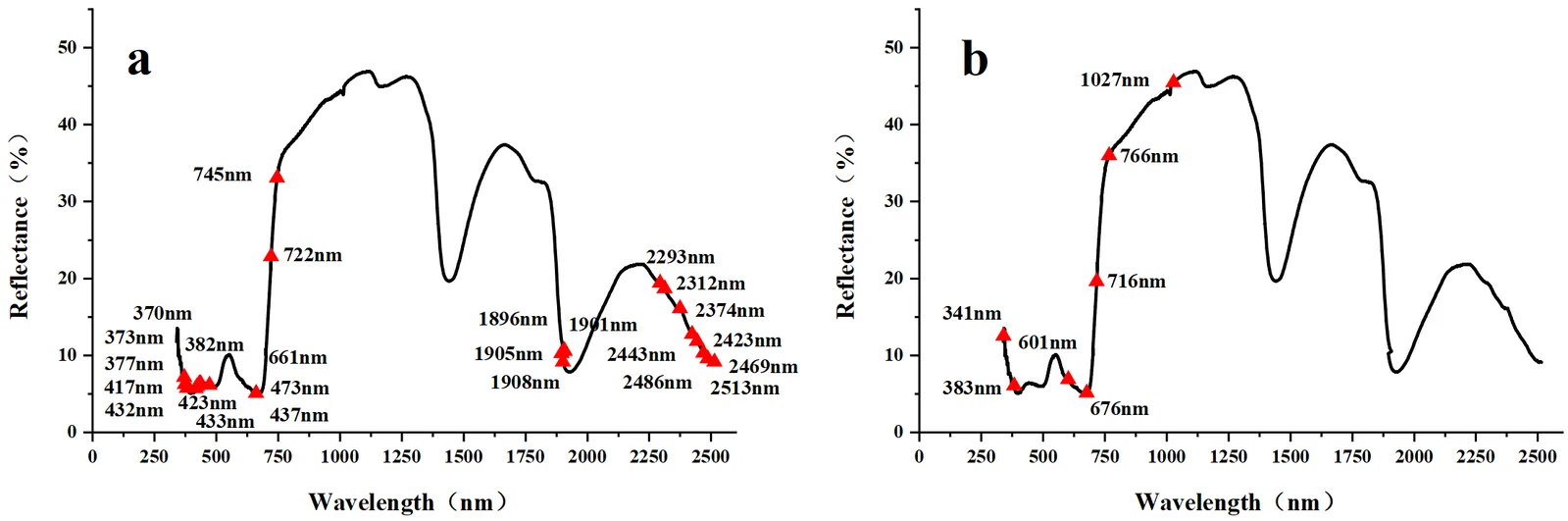
Band locations for feature extraction of raw reflectance by CARS **(a)** and SPA **(b)**.

The number of features selected by the CARS algorithm is more than that selected by the SPA (**Figure 7**). Of the 7 sensitive bands selected by the SPA algorithm, 4 are in the visible band, which includes the strong absorption and reflection peaks of chlorophyll, directly reflecting the intensity of photosynthesis. In addition, two sensitive bands in the 700–770 nm range are also related to chlorophyll content, the reflectance rises sharply and has a steep, almost linear shape. The slope of this curve is directly influenced by the amount of chlorophyll (a+b) per unit area of the plant. Another selected band falls in the 1000–1300 nm range, where crop leaves have strong reflective properties. The varying pro-portions of the major components in the leaves due to experimental treatments cause this reflective capacity to vary in strength, resulting in noticeable differences in spectral reflectance in this band. Compared to the SPA algorithm, the bands selected by the CARS algorithm include bands in the mid-infrared band (1830–2080 nm, 2080–2350 nm, 2350–2500 nm) in addition to those in the visible spectrum. This region contains the strong absorption bands for water and carbon dioxide.

### 3.4 Model construction

**Figure 8:**
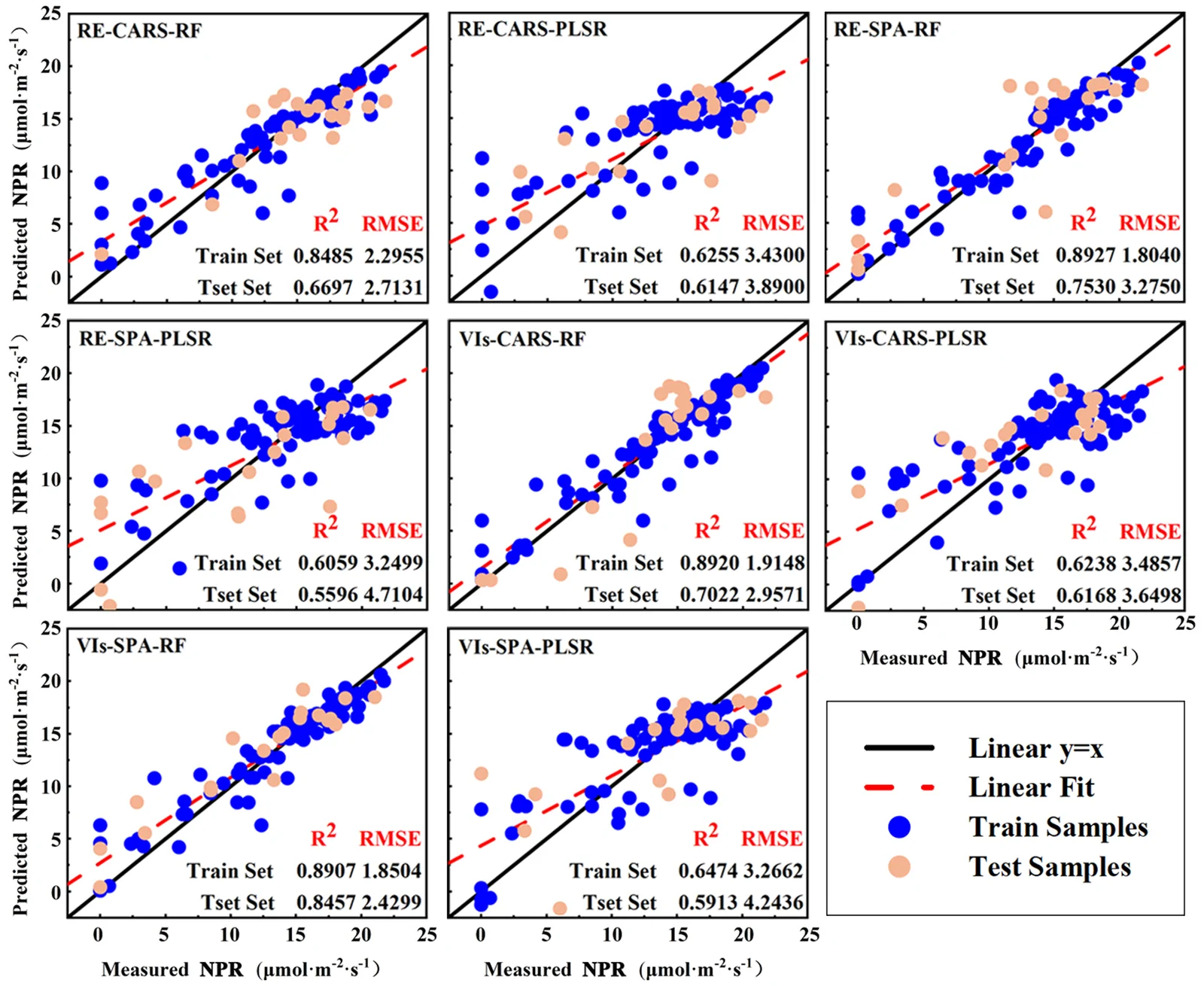
Distribution of measured and predicted values of estimation models to NPR.

Comparing the results of four different variable selection methods, models construct-ed using vegetation index combination performed better than those constructed using raw spectral reflectance. Furthermore, models constructed using the RF algorithm significantly outperformed those constructed using PLSR. Scatter plots of measured versus predicted values of NPR for different combinations of variable selection and modelling methods are shown in **Figure 8**.

Substantial performance differences were observed between RF models and PLSR models. For the RF model, the actual and predicted values of both the training and validation samples are largely clustered around the 1:1 line. The coefficient of determination exceeded 0.8 for the modelling set (R^2^_train) and 0.60 for the validation set (R^2^_test). In contrast, PLSR models exhibited greater dispersion around the 1:1 line, with R^2^ value ranging between 0.50 and 0.70 for both modelling and validation sets, indicating inferior ac-curacy and stability compared to the RF models. Among the four RF configurations, mod-els based on VI combinations achieved markedly higher R^2^ compared to the raw reflectance models, representing 4% improvement in modelling accuracy. However, this ad-vantage diminished in validation, with R^2^ decreasing by 11% compared to the raw reflectance models. The four PLSR models have relatively low accuracy, resulting in minor differences between them. Nevertheless, models constructed using VI combinations margin-ally outperformed those based on raw spectral reflectance.

In addition, each scatterplot contained outliers at x=0 that deviated substantially from the 1:1 line. These outliers originated from winter wheat samples at the late grain-filling stage (21 and 28 DAA). Different water and nitrogen application rates led to accelerated developmental processes in some treatments, resulting in an early end to grain filling and entry into the ripening phase. This resulted in a sharp decline in growth activity, with the actual measured NPR being zero. However, these samples had predicted values and therefore did not fit the distribution pattern of y=x. Each model contains 6 such outliers, representing 6% of the total experimental samples, and they are distributed in both the modelling and validation sets, having a small impact on both. Considering that this situation is an inevitable phenomenon resulting from the combined effects of drought stress and nitrogen deficiency on winter wheat plants, these outliers were retained in the analysis.

The stage-specific analysis showed that the optimal model maintained relatively stable performance from DAA1 to DAA21, with R² values ranging from 0.85 to 0.90 (**Table 3**). The highest accuracy was observed at DAA1 (R² = 0.90), which was slightly higher than the overall model performance (R² = 0.8896). In contrast, model performance declined markedly at DAA28, where R² decreased to 0.64 (ΔR² = −0.2496) and RMSE increased to 2.99, representing a 239.3% increase relative to the overall RMSE. Nitrogen-specific analysis further revealed that prediction accuracy decreased progressively with nitrogen deficiency. The lowest accuracy occurred under N0 (R² = 0.78; RMSE = 2.38), followed by N1 (R² = 0.83; RMSE = 1.91). Compared with the overall model, RMSE increased by 170.0% under N0 and by 116.7% under N1. By contrast, the model performed best under moderate-to-high nitrogen supply, particularly under N4 (R² = 0.93; RMSE = 1.39). These results quantitatively demonstrate that the major prediction degradation occurred during late senescence (DAA28) and under severe nitrogen deficiency (N0–N1).

**Table 3:** Stage- and nitrogen-specific prediction performance of the optimal model.

Factor	R²	Bias	RMSE	Bias
DAA1	0.9	+0.0104	1.39	-0.4384
DAA7	0.85	-0.0396	1.57	-0.2584
DAA14	0.87	-0.0196	1.37	-0.4584
DAA21	0.89	+0.0004	1.73	-0.0984
DAA28	0.64	-0.2496	2.99	+1.1616
N0	0.78	-0.1096	2.38	+0.5516
N1	0.83	-0.0596	1.91	+0.0816
N2	0.86	-0.0296	1.96	+0.1316
N3	0.91	+0.0204	1.55	-0.2784
N4	0.93	+0.0404	1.39	-0.4384

Bias represents the change in performance for each sample set and all samples of the optimal model.

### 3.5 Model validation

**Figure 9:**
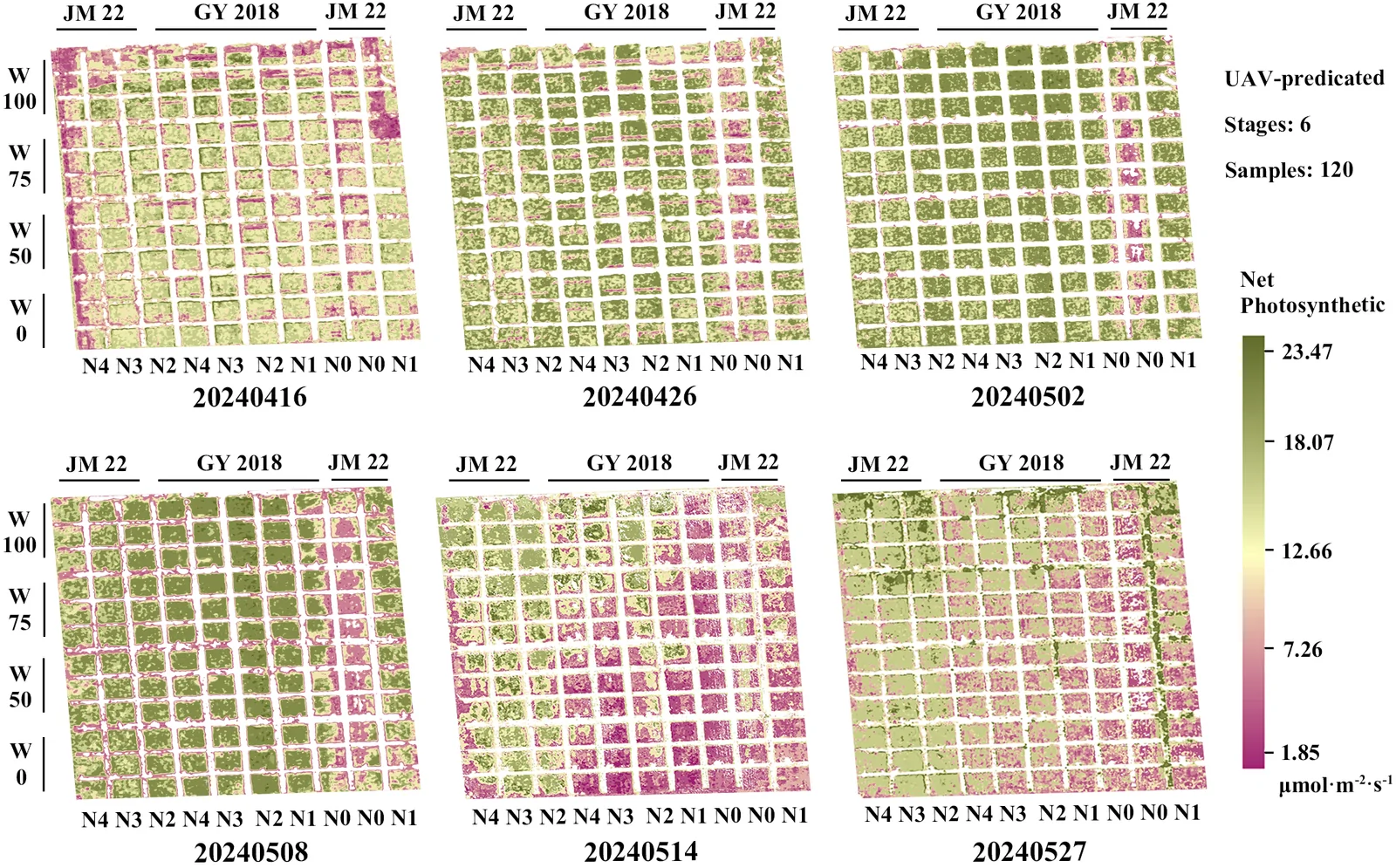
Field scale spatial distribution of predicted NPR in exp.2.

After effective cross-platform calibration, the UAV-based system was able to apply the model trained with field spectroradiometer data to provide reliable predictions of NPR. Overall, the predicted NPR values were consistent with the physiological range of winter wheat and successfully captured inter sample variability (**Figure 9**). This variability was mainly reflected in three aspects. First, clear differences were observed across growth stages. From 16 April to 8 May, the overall NPR level increased across most plots, and a larger proportion of plots showed medium-to-high NPR values. Notably, the prediction accuracy did not fully extend to some treatments at maturity. After 8 May, plots with medium to high NPR levels (16.00-20.00) continued to show a decreasing trend, whereas plots with low NPR levels (1.00-8.00) exhibited an increasing trend, indicating reduced prediction consistency under these conditions. Second, the predicted maps showed distinct responses to water and nitrogen treatments. Under the same growth stage, plots receiving higher nitrogen levels generally exhibited higher predicted NPR values than low-nitrogen plots. Differences among irrigation treatments were also detectable, although the contrast was less pronounced than that observed among nitrogen treatments. Third, varietal differences in NPR were effectively identified. Under the same growth stage and identical water and nitrogen supply conditions, the model distinguished NPR levels between two winter wheat cultivars. These differences were less pronounced at moderate NPR levels (10.00-16.00) but became evident at low and high NPR ranges. In particular, when both cultivars were under severe nitrogen deficiency, the differences in predicted NPR were especially pronounced.

## 4 Discussion

### 4.1 The impact of water and nitrogen supply on NPR and spectral reflectance

Photosynthesis is a complex physiological prepress highly sensitive to environmental factors. Water and nitrogen availability directly affects the water and nitrogen content of plants, consequently altering the NPR. Regarding irrigation, the NPR exhibited a positive linear response to water supply. This relationship arises because drought stress attenuates overall plant physiological activity, impairing the functionality of metabolic enzymes essential for physiological processes (Supratim et al., 2016), and severe drought stress can even lead to plant death. In terms of nitrogen application, increasing nitrogen supply generally increases the nitrogen content of plants and soils, whereas nitrogen-deficient conditions reduce nitrogen availability and constrain physiological activities such as photosynthesis and leaf growth (Zörb et al., 2018). Under low-nitrogen treatments (N0–N1), limited nitrogen availability restricted the synthesis of chlorophyll and photosynthetic enzymes, including Rubisco, resulting in reduced photosynthetic capacity and lower NPR. As nitrogen supply increased to moderate levels (N2–N3), leaf nitrogen status improved, photosynthetic machinery developed more fully, and NPR increased accordingly. However, further nitrogen addition did not lead to a continued increase in NPR. In the N4 treatment, excessive nitrogen supply may have promoted excessive vegetative growth and increased canopy shading, thereby reduced light-use efficiency and limited further improvement in photosynthetic performance. Consequently, NPR in N4 was slightly lower than that in N3.

In terms of the effect on leaf spectral properties, different water and nitrogen application rates determine leaf water and nitrogen content, which in turn affects the absorption and reflection of light energy (Daughtry et al., 2010). In the visible and shortwave infrared bands, reflectance decreases with increasing water content, whereas the opposite occurs in the near infrared band (Carter, 1993). Reflectance in the visible band decreases with increasing nitrogen content, while the opposite is observed in the NIR and shortwave NIR bands (Ayala-Silva and Beyl, 2005). In this study, the varying water (nitrogen) content in leaves is a direct reflection of the different water (nitrogen) supply conditions. With the increase of irrigation amount, leaf water content increases. With the increase of nitrogen application, leaf nitrogen content first increases (N0-N3) and then decreases (N3-N4). The observed patterns of changes in spectral characteristics are consistent with the results of previous studies (Hinzman et al., 1984; Walsh et al., 2020).

### 4.2 Estimation of NPR

In this study, both leaf hyperspectral reflectance and field-measured NPR covered a range of different water and nitrogen application rates and different growth stages. With a total of 100 experimental samples included, the number of samples is considered sufficient for the purposes of the study. Most outliers were concentrated under severe drought and low-nitrogen conditions and during late senescence, where NPR approached zero and spectral responses became highly heterogeneous. These observations suggest that the retained outliers represent biologically meaningful extreme-stress samples rather than measurement errors, highlighting the increased uncertainty of hyperspectral NPR estimation under combined severe water and nitrogen stress.

Regarding feature selection, the SPA demonstrated superior dimensionality reduction compared to the CARS algorithm. The SPA achieved greater reduction and a more accurate estimation model. The SPA algorithm selected 7 sensitive bands distributed in the visible band, the 700–770 nm band, and the 1000–1300 nm band. The visible band contains the strong absorption band and a strong reflection peak of chlorophyll, which is a primary pigment in photosynthesis and plays a central role in light absorption. Therefore, changes in spectral reflectance within this range can directly reflect the intensity of photosynthesis. The reflectance spectrum of crop leaves in the 700–770 nm band is also closely related to chlorophyll content. The reflectance spectrum rises sharply, showing a steep and nearly linear shape, with its slope directly affected by the chlorophyll (a+b) content per unit area of the plant (Ramos et al., 2020). Additionally, in the 1000–1300 nm band, crop leaves exhibit strong reflectance characteristics. As this study was conducted in the field, measurements were taken at noon when atmospheric temperatures are high. The leaves initiate a self-defense mechanism against scorching, as noted (Carter, 1993). The reflectance spectrum at this band shows notable differences due to the varying strength of this defense capability resulting from experimental treatments. In comparison to the SPA, the CARS algorithm selects bands not only in the visible band but also in the mid-infrared band (1830–2080 nm, 2080–2350 nm, 2350–2500 nm), which includes strong absorption bands for water and carbon dioxide. The mid-infrared band is highly sensitive to water content. Previous studies have frequently used mid-infrared spectral reflectance to measure soil moisture or plant water content (Sims and Gamon, 2003; Fabre et al., 2015; Jing et al., 2019). However, this study did not exclude data on water and carbon dioxide, which are essential raw materials for photosynthesis in winter wheat and directly impact the NPR. The results indicate that the model’s accuracy was not affected by the inclusion of this data. In summary, the CARS and SPA algorithms achieved dimensionality reductions of 2.4% and 0.7%, respectively, in the raw spectral reflectance. The SPA algorithm compressed the input variables to a greater extent.

In constructing models, transforming the raw spectral reflectance into vegetation indices obviously improves model accuracy. The model built using the RF method after vegetation index transformation shows the best performance. Models constructed with a combination of various vegetation indices perform better than those of single index in previous research (El-Hendawy et al., 2017; Tan et al., 2018). In terms of modeling methods, the PLSR model has the advantage of being able to significantly predict features arising from biophysical and biochemical activities (Heckmann et al., 2017), but it performs worse compared to RF in this study. The superiority of RF suggests that the relationship between hyperspectral reflectance and winter wheat NPR is not purely linear. NPR is jointly regulated by chlorophyll concentration, leaf nitrogen status, stomatal conductance, mesophyll activity, and canopy structure. These factors interact nonlinearly during crop development, especially under contrasting water and nitrogen conditions. Consequently, changes in spectral reflectance do not necessarily produce proportional changes in NPR. PLSR assumes an approximately linear relationship between latent spectral variables and the target variable, which limits its ability to capture threshold effects, saturation phenomena, and complex interactions among spectral bands. In contrast, RF is an ensemble learning algorithm based on multiple decision trees and does not require a predefined functional form between predictors and response variables. RF can effectively model nonlinear relationships, high-order interactions, and heterogeneous responses among spectral variables, while also being relatively robust to multicollinearity and noise. These characteristics are particularly advantageous for photosynthetic capacity estimation from hyperspectral data, where information from chlorophyll absorption bands, red-edge regions, and near-infrared structural bands jointly contributes to NPR prediction. Similar findings have been reported in previous studies showing that RF often outperforms linear methods for estimating crop physiological traits from hyperspectral observations (Endo et al., 2001; Ramos et al., 2020).

### 4.3 Limitations and perspective of the study

In this study, the selection of vegetation indices was based on extensive consideration of leaf structure, pigments and photosynthetically required products. The selected vegetation indices were all based on previous research and no new indices specifically tailored to the estimation of NPR s for winter wheat were created. Although the vegetation indices gave satisfactory results in this work, the consummate of the method still needs to be improved. The estimation effects of other vegetation indices will be further investigated to continuously improve the model in future. In addition, considering that the differences in NPR s are most pronounced during the grain-filling period, which is the latter part of the reproductive growth stage, all experimental data for this study were collected in May, within the grain-filling period. Validation across different growing seasons revealed several limitations in the applicability of the proposed model. In particular, noticeable prediction bias was observed under conditions of severe senescence or extreme nutrient deficiency. This limitation mainly arises from the fact that the NPR estimation model relies on vegetation indices as input features. Under such extreme conditions, vegetation indices fail to adequately represent crop growth status, as canopy signals weaken and soil background effects become dominant. To address this issue, two main directions are proposed for future research. First, more effective removal of soil background information from UAV hyperspectral imagery should be explored to reduce interference and enhance the functional sensitivity of vegetation indices under extreme growth conditions. Second, additional input features beyond vegetation indices, such as texture features, could be incorporated. These features may capture complementary structural information and remain informative even under advanced senescence. The pronounced decline in prediction accuracy at DAA28 is likely associated with crop senescence. During late senescence, chlorophyll degradation, leaf yellowing, structural collapse, and increased tissue heterogeneity substantially alter canopy spectral characteristics. Consequently, the spectral-response relationship established during active growth stages becomes less stable, leading to larger prediction errors. Nitrogen deficiency also significantly reduced model accuracy. Under N0 and N1 treatments, leaves exhibited lower chlorophyll content, reduced photosynthetic capacity, and altered pigment composition, which weakened the sensitivity of hyperspectral features to NPR. The consistent increase in RMSE under N0–N1 indicates that the model tended to overestimate NPR under severe nitrogen stress. Importantly, the model showed comparatively high and stable accuracy during DAA1–21 and under N2–N4 treatments. Therefore, the proposed transferable hyperspectral model is most suitable for high-throughput estimation of winter wheat NPR during the active photosynthetic period and under moderate-to-high nitrogen supply, whereas caution is required when applying the model during late senescence or under severe nitrogen deficiency. In contrast, irrigation-induced variations in NPR were less effectively captured, which may partially limit the model’s generalizability across different water management regimes. To improve robustness, future work will include targeted experiments with a wider range of irrigation treatments, aiming to strengthen the model’s ability to represent NPR variability driven by water availability.

## 5 Conclusions

This study focused on winter wheat under varying water and nitrogen supply conditions and developed a high-throughput model for estimating NPR by integrating field spectroradiometer and UAV-based hyperspectral data. Significant differences in NPR were observed under different irrigation and nitrogen treatments, providing diverse samples for model development. The results demonstrated that hyperspectral reflectance effectively captured inter-treatment variability, with each band exhibiting distinct spectral responses. For model construction, both the CRAS and SPA algorithms effectively reduced hyperspectral data dimensionality. SPA showed superior performance by compressing sensitive variables to only 0.7% of the original spectral bands, significantly reducing computational complexity. Furthermore, transforming reflectance into vegetation indices markedly improved model performance. The VIs-SPA-RF model achieved the best results, with an R^2^ of 0.8907 and RMSE of 1.8504 for the training set, and an R^2^ of 0.8457 and RMSE of 2.4299 for the validation set. To enable cross-platform application, spectral discrepancies caused by differences in observation scale between ground-based and UAV hyperspectral data were statistically characterized. Based on this, feature correction and vegetation index normalization were applied to UAV data. After eliminating these differences, UAV imagery could be directly input into the VIs-SPA-RF model, enabling accurate and high-throughput monitoring of NPR in winter wheat fields.

Overall, this study established a cross-scale transferable framework that integrates the accuracy of field spectroradiometers with the high-throughput capability of UAV platforms. The results confirm the feasibility of this approach. Beyond NPR estimation, the proposed framework also provides a reference for monitoring other crop growth parameters. Future work will incorporate additional data sources, such as thermal infrared and chlorophyll fluorescence, to further improve estimation accuracy.

## Data Availability

The raw data supporting the conclusions of this article will be made available by the authors, without undue reservation.
